# Cyst between Femoral Attachments of Cruciate Ligaments: Unusual Cause of Knee Pain and Review of Literature

**DOI:** 10.1155/2020/8833683

**Published:** 2020-07-25

**Authors:** Ravi Gupta, Akash Singhal, Rohil Mehta

**Affiliations:** ^1^Orthopaedics cum Project Director, Sports Injury Center, Government Medical College Hospital, Chandigarh, India; ^2^Government Medical College and Hospital, Chandigarh, India; ^3^Department of Orthopaedics, Government Medical College and Hospital, Chandigarh, India

## Abstract

**Introduction:**

Cysts associated with the cruciate ligaments are rare with an incidence of less than 1%. Most are asymptomatic and detected as incidental findings, but some are symptomatic presenting with knee pain and restricted range of motion. *Case report*. A 33-year-old female presented with pain and restriction of terminal flexion at the knee. No history of trauma was present. Clinically no diagnosis could be established. MRI showed a cyst located between the femoral attachments of cruciate ligaments which was further confirmed by diagnostic arthroscopy. Rupture of the cyst was done arthroscopically. Postoperatively, patient was relieved of the pain. There were no subsequent recurrences.

**Conclusion:**

Cysts between the cruciate ligaments must be kept as a differential while dealing with a patient of knee pain, with no antecedent history of trauma and where clinically no diagnosis can be made. Arthroscopic excision or rupture has a good success rate with no recurrences.

## 1. Introduction

Cysts within the knee are mostly found around ACL, but they are also seen in different locations like around posterior cruciate ligament (PCL), within the substance of ACL, infrapatellar fat pad, beneath medial meniscus, and over the lateral meniscus [1]. The first report of a cyst within the anterior cruciate ligament was made by Caan in 1924 [2]. It is unclear how or why these cysts originate, with some theories including displacement of synovium into the surrounding tissue, or degenerative and proliferative changes of pluripotent mesenchymal cells following trauma [1, 2]. One theory involves the cellular response to trauma that liberates a mucin substance, hyaluronic acid [3]. This is interspersed with the fibers of the ligament, causing its fusiform dilatation [4]. With joint and tissue motion, the mucin substance dissects the ligament fibers and may be found at the ligament attachments or in the intercondylar notch of the knee [3, 4]. Intra-articular ganglion cysts especially those arising from the cruciate ligaments are very uncommon, with a reported prevalence of 0.2%–1.3% on MR imaging and 0.1%–0.6% on knee arthroscopy [1]. Asymptomatic cysts do not cause the clinically dominant symptoms and are incidentally detected with other knee lesions [4]. Symptomatic cysts are responsible for pain and discomfort, occur without concomitant intra-articular pathologies [4]. We report a case of a 33-year-old female presenting with progressively increasing pain and restriction of knee flexion for 3 years. The MRI showed a cyst between the cruciate ligaments which was confirmed by arthroscopy.

## 2. Case Report

**Figure 1:**
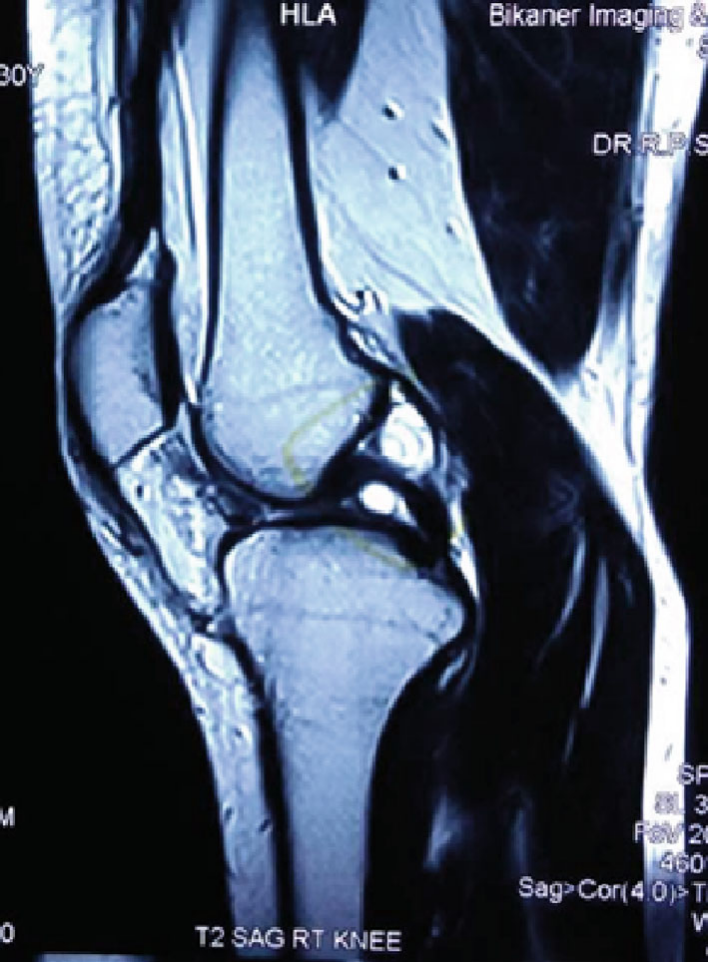
Saggital section of MRI showing cyst between the femoral attachment of two cruciate ligaments.

**Figure 2:**
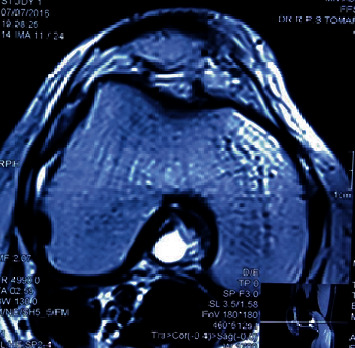
Axial section of MRI showing cyst between the femoral attachment of two cruciate ligaments.

**Figure 3:**
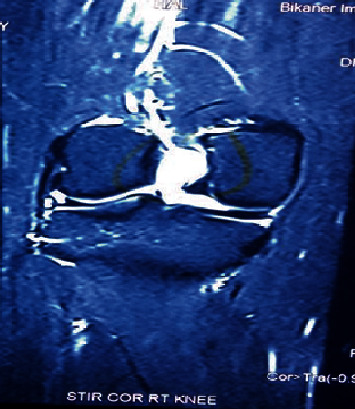
Coronal section of MRI showing cyst between the femoral attachment of two cruciate ligaments.

A 33-year-old female presented with chief complaint of pain and restriction of knee flexion since three years. Pain was gradual in onset, dull aching in character, progressively increasing over three years, increased on walking and relieved by rest. Patient had progressive limitation of flexion over three years. No history of antecedent trauma could be elicited. On examination, there was no palpable swelling. The tests for ligament laxity were negative. The range of knee motion was 0 to100 degrees and was terminally painful. We ordered MRI of the knee which showed a well-defined multilobulated cystic lesion in the intercondylar notch between the femoral attachments of cruciate ligaments measuring 17 × 12 × 10 mm (Figures 1–3). The ligaments were visualized as of normal intensity.

We planned arthroscopic surgery for the patient using the standard anterolateral and anteromedial portals. vIntraoperatively, a cyst was visualized between the anterior and posterior cruciate ligaments. The cyst wall was ruptured, and the clear fluid in the cyst was evacuated. The cyst had clear boundaries from the cruciate ligaments. Debridement of the cyst originating area with a power shaver was done. Postoperatively, the patient was started with range of motion exercises. The patient had complete relief of pain.

## 3. Discussion

Cysts associated with the cruciate ligaments are rare with Sarimo et al. and Mao et al. reporting an incidence of 0.36% on arthroscopy [5]. Krudwig et al. found the average age for the cyst as 31.2 years in 9 symptomatic patients, while Mao et al. found the average age of patients as 35.3 years [2, 5]. Mao et al. in their study of 31 patients also reported no knee trauma but suggested that repeated minor knee trauma contributed to the development of cyst based on the histological findings [5]. Krudwig et al. also advocated minor knee trauma to be responsible for development [2]. Garcia et al. also could not elicit any history of knee trauma in 8 out of 10 patients [6]. The pathogenesis is still unknown. For all the theories except one, the relationship to previous trauma is uncertain and has not been documented [7]. Yu et al. calculated the average age as 33.7 years in 12 patients [8]. Our patient was 33 years old, thus in the same age group with no history of knee trauma.

Most patients present with pain around the joint line, accompanied with some restriction in flexion or extension because of the worsening pain [1]. The incidence, severity, and duration of pain seem to vary depending on the size and location of the cyst [2]. Cysts located mainly anterior to cruciate ligaments tended to limit the extension of the knee, whereas those located predominately posterior to the cruciate ligaments tended to limit flexion [3]. It could be speculated that the changes in the length and torsion of the cruciate ligaments, due to knee motion, might result in traction or compression on the cysts that may stimulate the nerve endings on adjacent synovium and result in pain and abnormal sensation [9]. Garcia et al. reported pain to be associated with knee extension in 3 cases and with flexion in 3 cases [6]. However, most cases are incidental findings on arthroscopy and MRI without contributory symptoms. They are usually associated with other knee pathologies [10]. Garcia et al. reported cyst to be associated with ACL rupture in one case and meniscal lesions in four cases [6]. Krudwig et al. in their study of 85 cases reported the cysts to be associated with varying degrees of chondral damage and meniscal tears [2]. In 69 patients, there was Fairbanks III-IV chondromalacia of the medial femoral condyle, trochlea or patella, and macroscopic synovitis in 71 cases [2]. Concomitant lesions of the medial meniscus were reported in 42 patients, lateral meniscus in 17 patients, and of both the menisci in 13 patients [2].

MRI is the most sensitive and specific investigation to define the size and location of these cysts and to evaluate the joint for associated lesions [2, 4, 5]. The cysts appear as fluid-filled lobulated lesions having low signal intensity on T1 weighted images and high signal intensity on T2 images [2, 4]. Bergin et al. evaluated the coexistence of ACL cysts with mucoid degeneration on MRI [7]. Of the 4221 MRI examinations, 74 met the imaging criteria [7]. Out of the 74 MRI, 26 had features of both cysts and mucoid degeneration [7]. Both of these also had a high association with intraosseous ganglia thus representing a continuum of the degenerative process [7]. However, both of the conditions were not associated with ligament instability [7].

Arthroscopic cyst removal or rupture is the treatment of choice with a very low rate of recurrence [2, 5]. Mao et al. reported good results in 11 patients and Yu et al. in 12 patients using the standard anteromedial and anterolateral portals [2, 5]. Lunhao et al. showed good results in 16 cases and Parish et al in 15 cases with arthroscopy [11, 12]. Ultrasound, CT, or arthroscopic guided needle aspiration are associated with a higher rate of recurrence [2]. The prognosis after arthroscopic procedures is excellent as shown by Brown et al. with no recurrences [13, 14].

## 4. Conclusion

Cysts associated with the cruciate ligaments must be kept in mind when dealing with a patient with long-standing pain in the knee with normal clinical examination findings. The majority of the cases are asymptomatic with the symptomatic cases presenting with pain and restriction of knee motion. MRI is invaluable for diagnosis and the treatment of choice being arthroscopic debridement with no recurrences and satisfactory effects on the knee range of motion, which could increase the angiogenesis and collagen of the ACL and improve the proprioception of the knee.

## References

[1] Bui-Mansfield L. T., Youngberg R. A. (1997). Intra-articular ganglia of the knee: prevalence, presentation, etiology, and management. *AJR. American Journal of Roentgenology*.

[2] Krudwig W. K., Schulte K. K., Heinemann C. (2004). Intra-articular ganglion cysts of the knee joint: a report of 85 cases and review of the literature. *Knee Surgery, Sports Traumatology, Arthroscopy*.

[3] Bergin D., Morrison W. B., Carrino J. A., Nallamshetty S. N., Bartolozzi A. R. (2004). Anterior cruciate ligament ganglia and mucoid degeneration: coexistence and clinical correlation. *AJR. American Journal of Roentgenology*.

[4] Kakutani K., Yoshiya S., Matsui N., Yamamoto T., Kurosaka M. (2003). An intraligamentous ganglion cyst of the anterior cruciate ligament after a traumatic event. *Arthroscopy*.

[5] Zantop T., Rusch A., Hassenpflug J., Petersen W. (2003). Intra-articular ganglion cysts of the cruciate ligaments: case report and review of the literature. *Archives of Orthopaedic and Trauma Surgery*.

[6] Garcia A.-F., Garcia Pequerel J. M., Avila J. L., Sainz J. M., Castiella T. (2000). Ganglion cysts associated with cruciate ligaments of the knee: A possible cause of recurrent knee pain. *Acta Orthopaedica Belgica*.

[7] Ahluwalia V. V., DayanandaSagar G., Narayan S., Gupta A. (2014). Intercondylar ganglion cyst with mucoid degeneration of posterior cruciate ligament of knee: report of a rare case and review of literature. *Journal of Orthopedic Case Reports*.

[8] Yu H. C., Wen H., Zhang Y. (2014). Arthroscopic treatment of symptomatic anterior cruciate ligament cysts of the knee. *Zhongguo Gu Shang*.

[9] Plotkin B., Agarwal V. K., Varma R. (2009). Ganglion cyst of the anterior cruciate ligament. *Radiology Case Reports*.

[10] Maffulli N., Binfield P. M., King J. B. (1993). Isolated ganglions of the anterior cruciate ligament. *Medicine and Science in Sports and Exercise*.

[11] Lunhao B., Yu S., Jiashi W. (2011). Diagnosis and treatment of ganglion cysts of the cruciate ligaments. *Archives of Orthopaedic and Trauma Surgery*.

[12] Parish E. N., Dixon P., Cross M. J. (2005). Ganglion cysts of the anterior cruciate ligament: A series of 15 cases. *The Journal of Arthroscopic and Related Surgery*.

[13] Qi W., Wang J. L., Qu F., Li S. Y., Liu C., Liu Y. J. (2013). Arthroscopic reconstruction of anterior cruciate ligament with preservation of the remnant bundle. *Zhongguo Gu Shang*.

[14] Brown M. F., Dandy D. J. (1990). Intra-articular ganglia in the knee. *Arthroscopy*.

